# Attention‐aware 3D U‐Net convolutional neural network for knowledge‐based planning 3D dose distribution prediction of head‐and‐neck cancer

**DOI:** 10.1002/acm2.13630

**Published:** 2022-05-09

**Authors:** Alexander F. I. Osman, Nissren M. Tamam

**Affiliations:** ^1^ Department of Medical Physics Al‐Neelain University Khartoum Sudan; ^2^ Department of Physics College of Science Princess Nourah bint Abdulrahman University Riyadh Saudi Arabia

**Keywords:** 3D dose prediction, attention‐gated U‐Net, convolutional neural networks, deep learning, head‐and‐neck cancer, intensity‐modulated radiation therapy, knowledge‐based planning, radiation therapy, radiotherapy treatment planning

## Abstract

**Purpose:**

Deep learning–based knowledge‐based planning (KBP) methods have been introduced for radiotherapy dose distribution prediction to reduce the planning time and maintain consistent high‐quality plans. This paper presents a novel KBP model using an attention‐gating mechanism and a three‐dimensional (3D) U‐Net for intensity‐modulated radiation therapy (IMRT) 3D dose distribution prediction in head‐and‐neck cancer.

**Methods:**

A total of 340 head‐and‐neck cancer plans, representing the OpenKBP—2020 AAPM Grand Challenge data set, were used in this study. All patients were treated with the IMRT technique and a dose prescription of 70 Gy. The data set was randomly divided into 64%/16%/20% as training/validation/testing cohorts. An attention‐gated 3D U‐Net architecture model was developed to predict full 3D dose distribution. The developed model was trained using the mean‐squared error loss function, Adam optimization algorithm, a learning rate of 0.001, 120 epochs, and batch size of 4. In addition, a baseline U‐Net model was also similarly trained for comparison. The model performance was evaluated on the testing data set by comparing the generated dose distributions against the ground‐truth dose distributions using dose statistics and clinical dosimetric indices. Its performance was also compared to the baseline model and the reported results of other deep learning‐based dose prediction models.

**Results:**

The proposed attention‐gated 3D U‐Net model showed high capability in accurately predicting 3D dose distributions that closely replicated the ground‐truth dose distributions of 68 plans in the test set. The average value of the mean absolute dose error was 2.972 ± 1.220 Gy (vs. 2.920 ± 1.476 Gy for a baseline U‐Net) in the brainstem, 4.243 ± 1.791 Gy (vs. 4.530 ± 2.295 Gy for a baseline U‐Net) in the left parotid, 4.622 ± 1.975 Gy (vs. 4.223 ± 1.816 Gy for a baseline U‐Net) in the right parotid, 3.346 ± 1.198 Gy (vs. 2.958 ± 0.888 Gy for a baseline U‐Net) in the spinal cord, 6.582 ± 3.748 Gy (vs. 5.114 ± 2.098 Gy for a baseline U‐Net) in the esophagus, 4.756 ± 1.560 Gy (vs. 4.992 ± 2.030 Gy for a baseline U‐Net) in the mandible, 4.501 ± 1.784 Gy (vs. 4.925 ± 2.347 Gy for a baseline U‐Net) in the larynx, 2.494 ± 0.953 Gy (vs. 2.648 ± 1.247 Gy for a baseline U‐Net) in the PTV_70, and 2.432 ± 2.272 Gy (vs. 2.811 ± 2.896 Gy for a baseline U‐Net) in the body contour. The average difference in predicting the *D*_99_ value for the targets (PTV_70, PTV_63, and PTV_56) was 2.50 ± 1.77 Gy. For the organs at risk, the average difference in predicting the $D_{max}$ (brainstem, spinal cord, and mandible) and $D_{mean}$ (left parotid, right parotid, esophagus, and larynx) values was 1.43 ± 1.01 and 2.44 ± 1.73 Gy, respectively. The average value of the homogeneity index was 7.99 ± 1.45 for the predicted plans versus 5.74 ± 2.95 for the ground‐truth plans, whereas the average value of the conformity index was 0.63 ± 0.17 for the predicted plans versus 0.89 ± 0.19 for the ground‐truth plans. The proposed model needs less than 5 s to predict a full 3D dose distribution of 64 × 64 × 64 voxels for a new patient that is sufficient for real‐time applications.

**Conclusions:**

The attention‐gated 3D U‐Net model demonstrated a capability in predicting accurate 3D dose distributions for head‐and‐neck IMRT plans with consistent quality. The prediction performance of the proposed model was overall superior to a baseline standard U‐Net model, and it was also competitive to the performance of the best state‐of‐the‐art dose prediction method reported in the literature. The proposed model could be used to obtain dose distributions for decision‐making before planning, quality assurance of planning, and guiding‐automated planning for improved plan consistency, quality, and planning efficiency.

## 1 INTRODUCTION

The modern radiotherapy treatment techniques, such as intensity‐modulated radiation therapy (IMRT) and volumetric arc radiation therapy (VMAT) modalities, offer high‐dose conformity and submillimeter spatial precision. These characteristics have allowed physicians additional flexibility to maximize local tumors control by delivering a high dose to the planning target volumes (PTVs) while minimizing toxicities to normal tissues by sparing the adjacent critical structures or organs at risk (OARs).^1^, ^2^, ^3^ Conversely, the planning procedure of these techniques is technically demanding (e.g., requires significant knowledge and domain expertise) and time‐consuming.^4^

The workflow of the treatment planning of IMRT and VMAT techniques involves iterative trial‐and‐error processes until the set clinical criteria/goals are achieved (e.g., the PTVs dose coverage and OARs dose constraints). Given the patient‐computed tomography (CT) image data, contoured structures of PTVs and OARs, and prescription dose, a human planner solves an inverse optimization problem on a treatment planning system by progressively adjusting several optimization hyper‐parameters (e.g., locations and weights of dose–volume histogram (DVH) constraints). The goal of this tuning is to control the tradeoffs between clinical objectives based on the planner's experience to achieve a clinically satisfactory plan with optimal dose distribution meeting‐specific criteria. Because each patient's case is unique, it is challenging for the planner and physician to know in advance the achievable DVHs and the endpoint. The objective functions or dose constraints are usually defined by the planner according to standard clinical protocols, such as the quantitative analyses of normal tissue effects in the clinic guidelines.^5^ Considering the previous challenges, the plan quality and planning times determined by inverse planning are influenced by the skills and experience of planners and institutions and the available time.^6^, ^7^, ^8^ As a result, inconsistent and suboptimal plan dosimetry is common among different institutions and individual planners,^9^, ^10^, ^11^ resulting in a negative impact on tumor control in patients. Although generating a minimally acceptable plan may be quick, improving it is much more time‐consuming and can take hours or even days based on the complexity of the plan.^12^

As inverse planning experience and especially the carefully designed clinical plan data have accumulated throughout the past few decades, data‐driven knowledge‐based planning (KBP) methods have been introduced. In KBP, machine and deep learning algorithms are applied to learn patient geometrical anatomy and dose mapping on previous patient databases of high‐quality clinical acceptable treatment plans, then the trained model estimates optimal dose distributions for new patients. The treatment tradeoffs and clinician experience knowledge are assumed to be embedded in the design of previous clinical plans. The goal of the KBP approach is to improve the plan consistency, quality, and planning efficiency. In conventional KBP software algorithms for example, RapidPlan in Eclipse (Varian Medical Systems, Palo Alto, CA, USA), the DVHs are predicted from a group of previous plans and used as objectives to perform the inverse planning optimization for a new plan.^13^, ^14^, ^15^, ^16^, ^17^, ^18^ However, this suffers from a lack of spatial dose information within the contoured structures (e.g., locations of hot/cold spots, which a physician often pays attention to) as it only predicts the DVH objectives, which is essential for evaluating plan quality. It also requires manual feature designing and cannot support the automation of all plans.^19^, ^20^, ^21^ Handcrafted features on the patient plan cannot cover all inherent structure characteristics and only capture low‐level features, so the model will not be sufficiently accurate for dose prediction.^21^ On the other hand, contemporary KBP algorithms, which use the state‐of‐the‐art deep learning techniques, predict more accurate and robust full three‐dimensional (3D) dose distributions (which are used to generate post‐optimization deliverable plans).^22^, ^23^, ^24^, ^25^, ^26^, ^27^ DVH can be fully reconstructed from the predicted 3D dose distributions, and the dose constraints can then be calculated. Generating deliverable treatment plans involves calculations of the multileaf collimator leaf motion sequence to ensure that the predicted dose distributions satisfy the physical delivery constraints imposed by the linac.

Deep learning convolutional neural networks are capable of hierarchically capturing multiscale structural features directly from the input raw image data on their own. This characteristic resolves the limitations associated with the handcrafting features for individual works. Therefore, they can use all information available in the images. Over the past few years, deep learning techniques have rapidly grown and demonstrated remarkable performance with successful implementations in many fields, including radiation therapy.^12^, ^28^, ^29^, ^30^

Compared with IMRT planning of other anatomical sites, head‐and‐neck treatment planning is one of the most challenging sites requiring a high level of knowledge, human clinical expertise, and effort to produce high‐quality plans. These challenging aspects are due to the large size of the PTV, multiple prescription dose levels that are simultaneously integrated boosted, and the presence of several critical OARs nearby the PTV.^31^, ^32^ Several studies assessed various deep learning convolutional network architectures for predicting IMRT/VMAT dose distributions in head‐and‐neck cancer given the patient's anatomical information (CT scan only or/and contour structures). These methods include residual network,^33^, ^34^ hierarchically densely connected U‐Net,^35^, ^36^ dilated convolutional based U‐Net,^37^ cascade U‐Net,^38^ residual U‐Net (Res U‐Net),^39^ generative adversarial networks (GANs),^22^, ^40^, ^41^ and conditional GAN.^42^ The reported results by those models were promising; however, there is still a need for investigating other methods for better prediction accuracy and generalizability. The prediction model must be accurate because the quality of the final plans strongly correlates with the quality of the predictions.^43^

The attention‐gating mechanism has recently emerged to enable networks to highlight important anatomy features and suppress redundant information propagation through the network.^44^ It also helps encourage compatibility between the output function and the extracted intermediate local feature vectors in each network. Kearney et al.^45^ implemented attention‐gated GAN for dose distribution prediction for prostate cancer. Their results were inspiring by predicting more realistic dose distributions compared to the other state‐of‐the‐art algorithms. However, GANs are difficult to train and rely on compromised architectures to facilitate convergence. This study proposes a novel KBP method using 3D U‐Net with attention gates to improve its learning for predicting a full 3D dose distribution in head‐and‐neck cancer from 3D patient anatomical information. Attention‐gated convolutions technique can help to improve the prediction performance by reducing the network's redundancy to focus only on the more relevant anatomy that improves the learning. The 3D structure of the proposed model in this study allows considering the context between the adjacent image slices for more accurate predictions.

## 2 MATERIALS AND METHODS

### 2.1 Patient data

A total of 340 oropharyngeal/head‐and‐neck cancer patients who received simultaneous integrated boost IMRT were included in this study. The data were obtained from multiple institutions for the American Association of Physicists in Medicine (AAPM) Open Knowledge‐Based Planning Grand Challenge (OpenKBP—Grand Challenge).^46^ The planning data for each patient consisted of tensors of CT image, contoured structures (which describe the region, shape, and size of PTVs and OARs), and dose distributions (in Gy). The plans were generated and delivered with the IMRT step‐and‐shoot delivery technique using nine equally spaced coplanar beams of 6‐MV radiation energy. The clinical intent (dose prescription regime) was to deliver 70 Gy to the gross disease (PTV_70), 63 Gy to intermediate‐risk target volumes (PTV_63), and 56 Gy to elective target volumes (PTV_56) in 35 fractions. Each plan had at least one PTV (a PTV_70). The contoured PTV structures had no overlap with each other. The delineated OAR structures included the brainstem, left parotid, right parotid, spinal cord, mandible, esophagus, larynx, and external body contour. The delineation of the PTVs and OARs was performed by clinicians. All contours, CT images, and dose distributions tensors were down‐sampled to 128 × 128 × 128 voxels. The CT scans data were acquired with different resolutions with an average voxel size of 3.5 × 3.5 × 2 mm^3^.

### 2.2 Data preparation

Data preprocessing is a fundamental step in the workflow of network training. It may be more important than choosing the network itself for accurate predictions. We performed multistep processing before feeding the input data into the network. *First*, we cropped down the data size to a dimension of 64 × 64 × 64 voxels rather than 128 × 128 × 128 to minimize unimportant background area (i.e., by including only brain region) and avoid GPU/CPU memory overflow. *Second*, all OAR contoured structures were given as binary masks, where voxels inside the structure volume were assigned to 1 and 0 otherwise. A missing target (PTV_63 or PTV_56) or OAR mask in a patient planning data was controlled by creating a tensor of 64 × 64 × 64 voxels filled with zeros to maintain a complete set of data for each patient. *Third*, we performed data normalization to the input and target response data for each patient. The CT data were clipped/truncated to [0, 4095] HU range then normalized to [0, 1] range, whereas the ground‐truth dose distribution data were normalized to the prescription dose (70 Gy) before the training. It has been reported that intensity normalization helps to speed up the training and dose distribution normalization improves the predictions when CT and contours were used as input.^47^ Later as post‐processing, the predicted dose distribution was rescaled to the original prescription before the evaluation by multiplying by 70 (i.e., reverse normalization). *Finally*, we stacked the data of each patient to create a 12‐channel 3D positional information tensor of 64 × 64 × 64 as inputs to the network. The input data for each patient were arranged in a tensor of 12 channels as follows: (1) CT data, (2) PTV_70 mask, (3) PTV_63 mask, (4) PTV_56 mask, (5) brainstem mask, (6) left parotid mask, (7) right parotid mask, (8) spinal cord mask, (9) mandible mask, (10) esophagus mask, (11) larynx mask, and (12) body mask. This unique representation of the contours in distinct channels helps avoid the possibility of structures overlapping if they are all represented in a single channel due to their coarse voxel size. Lee et al.^48^ reported that the labeling order of OARs and PTVs does not affect the network performance and hence does not influence the prediction results.

### 2.3 Network architecture

**FIGURE 1:**
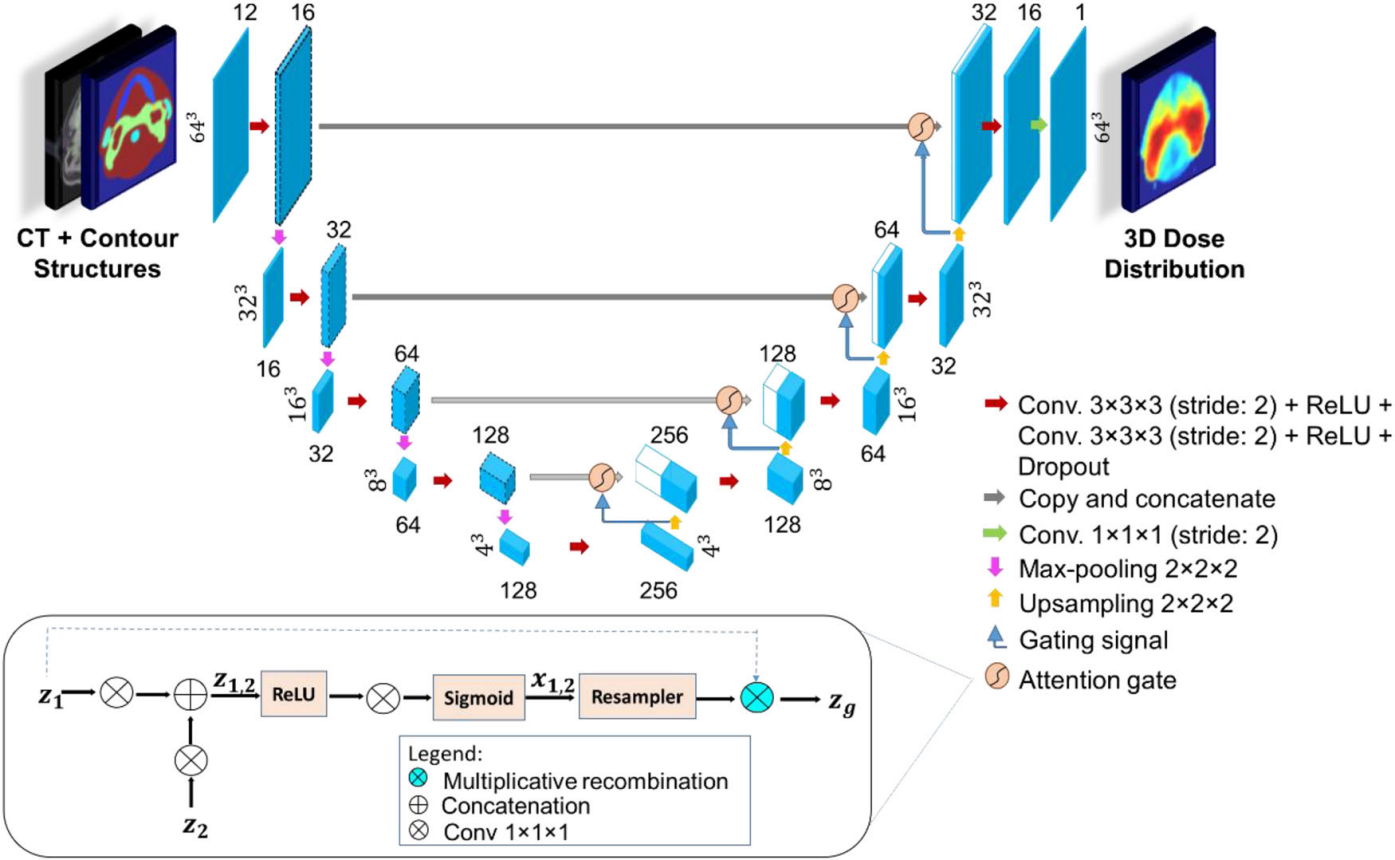
The attention‐gated 3D U‐Net architecture for KBP radiotherapy dose prediction. The patient anatomical information (CT and contour structures) are used as inputs to the model network to predict a full 3D dose distribution. Blue boxes correspond to a set of the feature map. The number of extracted feature maps is denoted on the top/bottom of the cubes. The size of the feature maps is provided at the left/right side of the box. White boxes represent copied feature maps. The arrows represent different operations. The attention gating mechanism is also shown in the figure for the propagation signal *z*_1_, gating signal *z*_2_, and fnal gated output signal $z_{g}$ for the network. CT, computed tomography; KBP, knowledge‐based planning

In this study, we extended the standard 2D U‐Net architecture^49^ to a 3D version for dose distribution prediction to account for the dependence on the anatomical geometry in the adjacent regions of the given anatomy. Then, we implemented the attention gates to the 3D U‐Net to selectively propagate information through a gating mechanism. These attention gates would make the network focuses on the relevant patterns on the input data. The architecture of the proposed attention‐gated 3D U‐Net model for 3D dose distribution prediction is demonstrated in Figure 1. It takes a 12‐channel 64 × 64 × 64 tensor as input data to predict the full 3D dose distribution.

The network architecture consists of five multiscale hierarchical levels made of a series of 3D convolutional layers. The encoder part contains two 3D convolutional layers (3 × 3 × 3 kernel size; voxel stride = 2) at each hierarchy level to extract a set of low‐ (voxel‐rich information) to high‐level (attribute‐rich information) features. In addition to feature extraction, these convolutional layers also find hierarchical representations over a wide receptive field of the 3D CT image and contour structures input data. Each convolutional layer in the encoder is followed by a rectified linear unit (ReLU) activation function.^50^ Zero padding was added to the convolution process to maintain the feature size constant. Dropout^51^ was used in the encoder after the second convolutional layer. The dropout rate increases in increments of 5% (from 10% to 30%) following each hierarchy level to mitigate overfitting and encourage the model to use all available filters within the network. These dropout rates were determined experimentally via a trial‐and‐error process, where the gap between the validation loss and training loss was minimal and did not tend to increase during training. A max‐pooling operation (2 × 2 × 2 kernel size; voxel stride = 2) was implemented between each two consecutive hierarchy levels to successively decrease the size of the feature maps (from 64^3^ to 4^3^), whereas the number of derived features simultaneously increase (from 16 to 256) by a factor of 2 at each level.

On the other hand, the decoder part is typically a mirrored version of the encoder. It continues to learn nonlinear relationships within the input data to reconstruct the targeted output tensor (that has the same resolution as the input) from the representation space in the network. The max‐pooling operation in the decoder is replaced with up‐sampling (2 × 2 × 2 kernel size; voxel stride = 2). The output at each hierarchy level in the encoder is concatenated to the corresponding one in the decoder through attention‐gated connections. The previous hierarchy level output in the decoder is used as the gating signal for the attention‐gated skip connections. The attention‐gating mechanism utilizes additive self‐attention gates to modulate multiscale level feature response propagation throughout each network.^52^ It implements a 3D convolution (1 × 1 × 1 kernel size; voxel stride = 1) to a propagation signal (*z*_1_) and a 3D convolution (1 × 1 × 1 kernel size; voxel stride = 2) to a gating signal (*z*_2_). Signals *z*_1_ and *z*_2_ are added together and the combined activations (*z*_1, 2_) are ReLU activated before being passed through a 1 × 1 × 1 convolutional kernel. The output is sigmoidally activated to form *x*_1, 2_. The final gated output signal ($z_{g}$) is formed by multiplying *z*_1_ by *x*_1, 2_.

The final layer in the network is a 3D convolutional layer (1 × 1 × 1 kernel size; voxel stride = 2) followed by a linear activation, yielding a voxel‐wise continuous output matching the input image resolution. The output is a 1‐channel 3D dose distribution matrix of a size of 64 × 64 × 64 voxels. The code for our 3D attention‐gated U‐Net model is publicly available at https://github.com/afiosman/attention-aware-3D-UNet-for-RT-dose-prediction.

### 2.4 Training and validating the model

Patients’ data were shuffled and divided into three disjoint cohorts: training set (64%: 218 plans), validation set (16%: 54 plans), and testing set (20%: 68 plans) to avoid multiple hypothesis testing. Before training the 3D attention‐gated U‐Net model from scratch, all trainable parameters (weights and biases) were initialized using the He et al.^53^ method. This method has shown better performance than other initialization methods for deep models with ReLU layers. The network was trained on the training data set to map the patients’ anatomical information (CT data and contour structures) to full 3D dose distributions for head‐and‐neck cancer. The voxel‐wise loss between the predicted dose $d_{predict}$ and the ground‐truth dose $d_{clinical}$ distribution during training is minimized through a mean‐squared error cost function as given in the following equation:

(1) $$\begin{array}{c} loss\;\left(predict,\;clinical\right)=\frac{1}{n}\;\sum_{i\;=\;1}^{n}{\left(d_{predict}^{i}-\;d_{ground\_truth}^{i}\right)}^{2}, \end{array}$$

where *i* is the index of the voxel and *n* is the total number of voxels. The training was performed using Adam stochastic optimization algorithm^54^ with the default parameter settings (*β*1 = 0.9, *β*2 = 0.999, and decay = 0) and a learning rate of 0.001 to regularly update the network trainable parameters after every epoch for improved prediction. The model was trained for 120 epochs with the training termination condition that was set to a 30‐epoch patience parameter. The batch size was set to four samples. The choice of this small batch size was due to the constraints of our memory and computational power. To prevent the likelihood of model overfitting, we implemented two regularization techniques: dropout (with increasing rates from 10% to 30%) and early‐stopping (patience parameter was set to a 30‐epoch) methods. During the model training, its performance was continuously validated on a hold‐out validation set of 54 patients to assess the training status. For a meaningful comparison of the proposed model performance to a baseline model, a standard U‐Net model (identical to the proposed one but without attention gates) was also similarly trained in this study on the same data set.

There were a total of 6,738,869 trainable parameters (weight matrices and biases) in the attention‐gated 3D U‐Net model. The training was executed on a computer with an Intel Core i5 processor (2.4 GHz) and an 8‐GB RAM. It took 43 h to train the model for 120 epochs. Once the model has successfully trained, it took only a few seconds to predict a full 3D dose distribution tensor of a size of 64 × 64 × 64 voxels for a new patient, making its clinical deployment possible. The model was implemented on Keras API (version 2.6) with a TensorFlow (version 2.6) platform as the backend in Python (version 3.7, Python Software Foundation, Wilmington, DE, USA).

### 2.5 Learning curve

**FIGURE 2:**
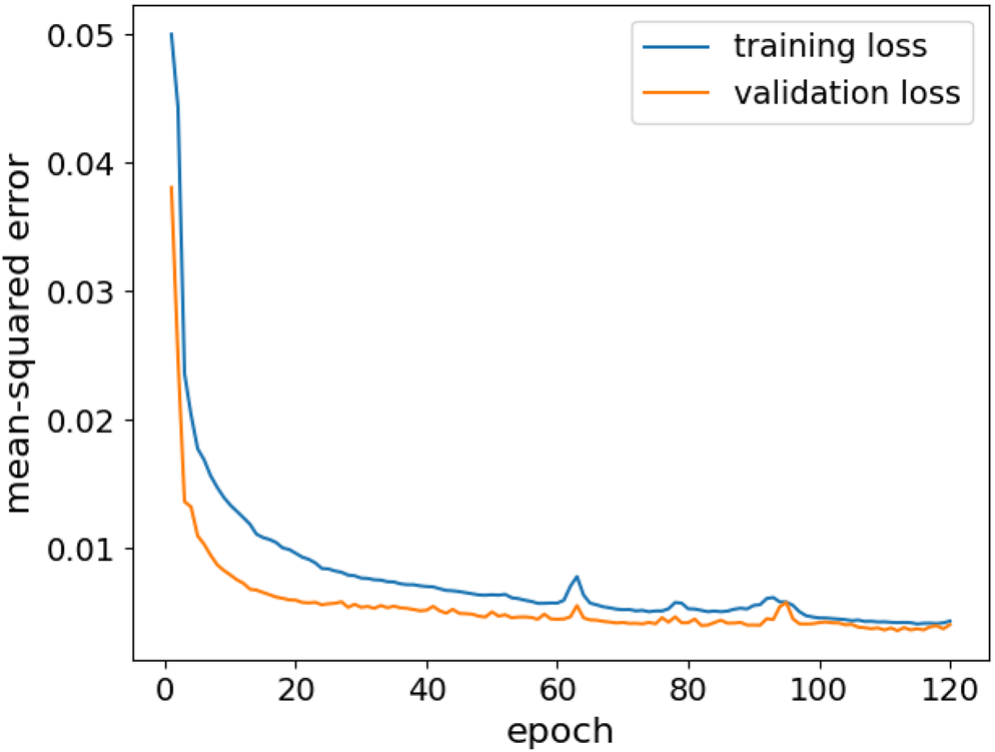
The learning curve of training loss versus validation loss over the number of epochs for the attention‐gated U‐Net model. The train learning curve gives an idea of how well the model is learning, whereas the validation learning curve highlights the model's generalizability

We analyzed the learning curve of the proposed model performance as shown in Figure 2 to identify whether the model has been sufficiently trained and learned the mapping. From the figure, we can notice that there is no indication of either model underfitting or overfitting. The training and validation losses can be seen progressively minimized over the model training until reaching convergence. The two curves, training and validation, have comparable performance exhibiting a good fit of our model for dose prediction and hence good generalization.

### 2.6 Evaluation

The model was evaluated on a testing cohort of 68 patients to assess its performance and generalizability for voxel‐wise volumetric dose predictions. The performance of the prediction model was assessed by comparing the predicted dose distributions against the ground‐truth dose distributions using voxel‐wise mean absolute error (MAE) and clinical‐related dosimetric indices. The formula of MAE is given as $MAE\;\left(x,y\right)=\frac{1}{n}\;\sum_{i\;=\;1}^{n}\left|y_{i}-x_{i}\right|$, where *n* is the total number of voxels, $x_{i}$ and $y_{i}$ are the ground‐truth and predicted dose values, respectively. To visually inspect the difference between the predicted and ground‐truth dose distributions, voxel‐wise difference map (residuals) was computed as follows: $\left(x_{i},y_{i}\right)=\;y_{i}-x_{i}$. The dose difference map helps quantify the errors in the predicted dose and provides spatial information (e.g., highlights the regions of high dose discrepancies and hot/cold spots). DVH indices concerning the OARs sparing and target coverage, for example, $D_{max}$, $D_{mean}$, and *D*_99_, were calculated to quantitatively evaluate the quality of the KBP plans against the ground‐truth plans. Furthermore, the homogeneity index (*HI*) and the conformity index (*CI*) were calculated for the target volume (PTV_70). The *HI* is defined as $HI\;=D_{2\%}\;-D_{98\%}$, where, $D_{2\%}$ and $D_{98\%}\;$are the percentage dose to 2% and 98% target volume.^55^ The *CI* is given as $CI\;=V_{RI}/TV$, where $V_{RI}$ and TV are the reference dose volume and the target volume, respectively.^56^ The proposed model performance was compared to a baseline standard 3D U‐Net. Furthermore, a rough comparison was performed between the prediction results in this study and that reported in the literature of other approaches presented at the Open‐KPB competition.

## 3 RESULTS

### 3.1 KBP prediction quality

**FIGURE 3:**
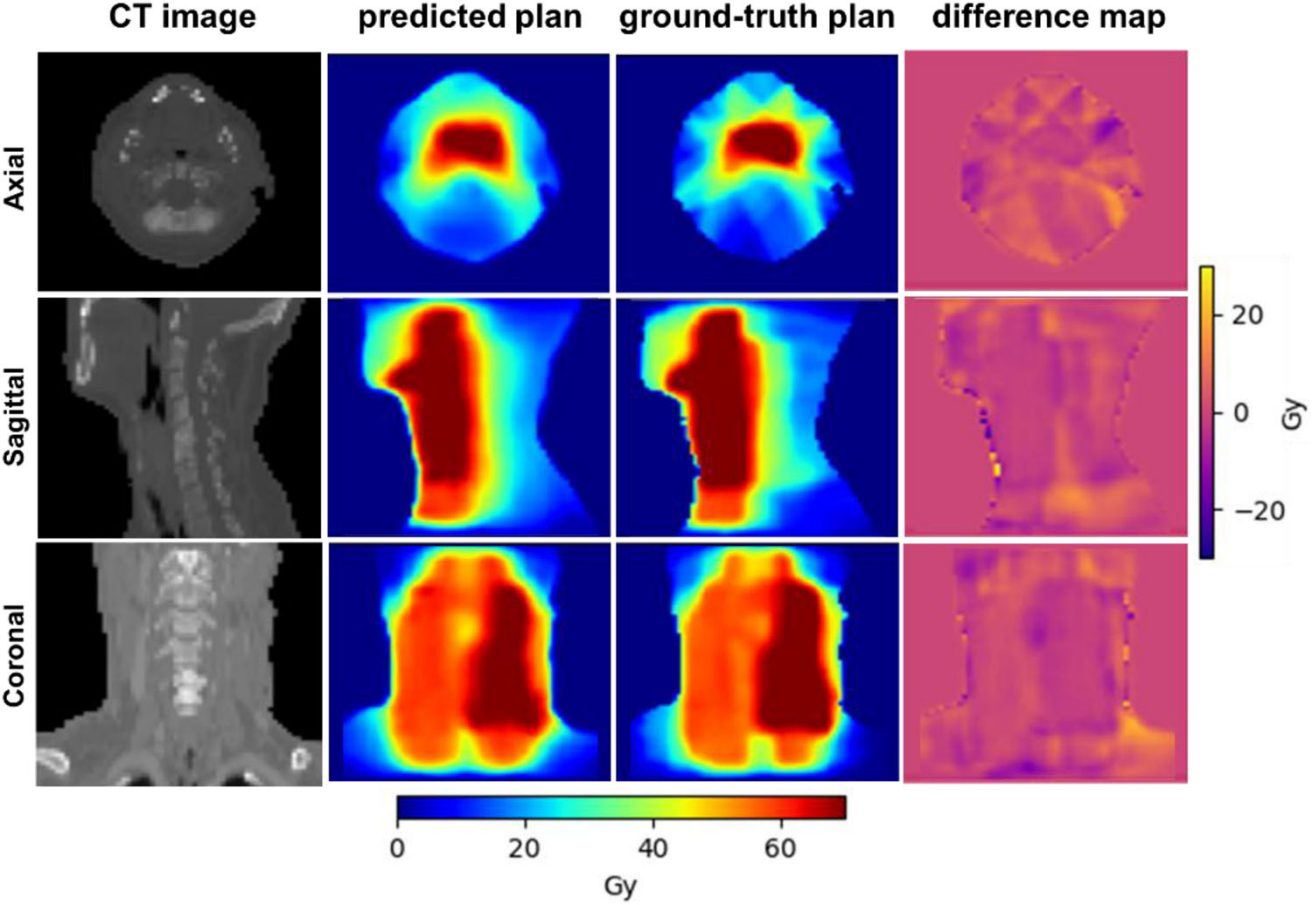
The CT image, KBP‐predicted dose distribution, ground‐truth dose distribution, and voxel‐wise dose difference map (predicted—ground‐truth) in the axial, sagittal, and coronal planes of a sample head‐and‐neck patient plan in the test set. CT, computed tomography; KBP, knowledge‐based planning

**FIGURE 4:**
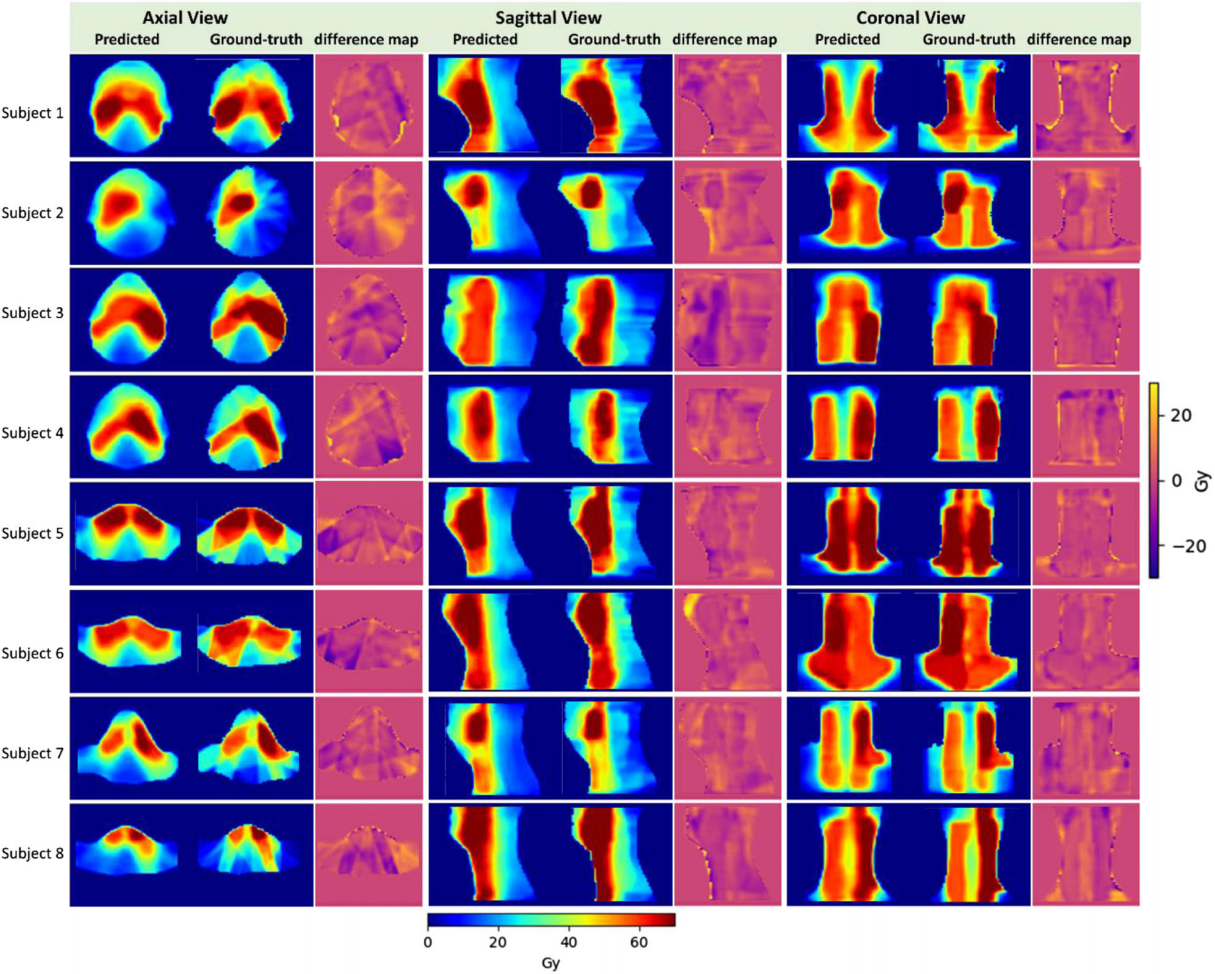
The predicted dose distributions were presented side‐by‐side with corresponding ground‐truth dose distributions in addition to difference maps for eight patients in the test set

The dose prediction results of the proposed attention‐gated 3D U‐Net model were presented in this section. Figure 3 shows an example of the predicted dose distributions displayed at different planes (axial, sagittal, and coronal) side‐by‐side with the ground‐truth dose distributions for comparison. The corresponding dose difference maps are depicted in the figure. The shape of the predicted dose distributions looked visually similar to the ground‐truth dose distributions. More examples of the prediction results are shown in Figure 4. From the figures, we can observe that the proposed model tended to produce slightly smoother dose distributions (e.g., smooth the steep dose gradient of IMRT) compared to the ground‐truth dose distributions. This is due to using coarse resolution (3.5 × 3.5 × 2 mm^3^) data to train the model. However, the model was capable of capturing the cold and hot spots in the plans.

**FIGURE 5:**
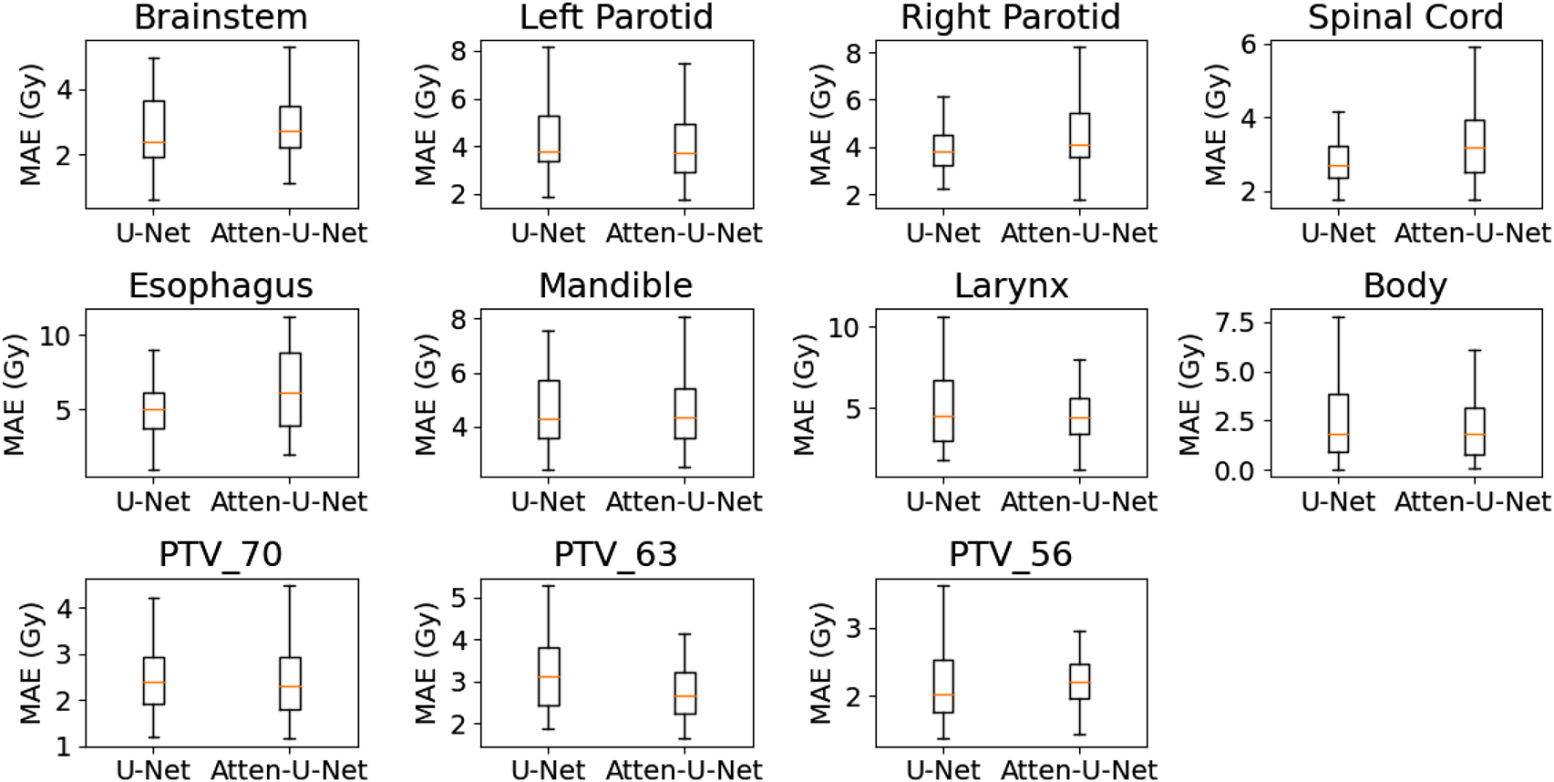
Boxplot of the MAE between the predicted and ground‐truth dose distributions for the targets and OARs contoured structures of all patients in the test set. The upper and lower boundaries of each box represent the 75th and 25th percentiles, respectively. The red line in the box depicts the median. Whiskers extend to 1.5 times the interquartile range and the most extreme outlier. MAE, mean absolute error; OAR, organs at risk

The error of predicted dose with the proposed model as well as the baseline model was quantified with respect to the ground‐truth dose using a voxel‐based MAE metric. This MAE was assessed in the PTVs, OARs, and globally in the body contour. The box plots in Figure 5 show the mean, median, and standard deviation of 3D dose MAE for the PTV and OAR regions for all patients (68 plans) in the test data set. The average MAE dose value in the body contour was 2.432 ± 2.272 Gy (attention U‐Net) versus 2.811 ± 2.896 Gy (baseline U‐Net). For the target volumes, the MAE value was 2.494 ± 0.953 Gy (attention U‐Net) versus 2.648 ± 1.247 Gy (baseline U‐Net) in the PTV_70, 2.904 ± 0.993 Gy (attention U‐Net) versus 3.513 ± 1.651 Gy (baseline U‐Net) in the PTV_63, and 2.327 ± 0.681 Gy (attention U‐Net) versus 2.251 ± 0.786 Gy (baseline U‐Net) in the PTV_56 structure. For the OARs, the value was 2.972 ± 1.220 Gy (attention U‐Net) versus 2.920 ± 1.476 Gy (baseline U‐Net) in the brainstem, 4.243 ± 1.791 Gy (attention U‐Net) versus 4.530 ± 2.295 Gy (baseline U‐Net) in the left parotid, 4.622 ± 1.975 Gy (attention U‐Net) versus 4.223 ± 1.816 Gy (baseline U‐Net) in the right parotid, 3.346 ± 1.198 Gy versus (attention U‐Net) 2.958 ± 0.888 Gy (baseline U‐Net) in the spinal cord, 6.582 ± 3.748 Gy (attention U‐Net) versus 5.114 ± 2.098 Gy (baseline U‐Net) in the esophagus, 4.756 ± 1.560 Gy (attention U‐Net) versus 4.992 ± 2.030 Gy (baseline U‐Net) in the mandible, and 4.501 ± 1.784 Gy (attention U‐Net) versus 4.925 ± 2.347 Gy (baseline U‐Net) in the larynx structure. The results show that dose distribution can be predicted within a 3.5% (relative to prescription dose) mean absolute dose error using the attention‐gated U‐Net model.

### 3.2 Dose–volume statistics

DVHs were used to evaluate the KBP plans to provide a clinical measure of prediction quality. The dose–volume indices were computed for both the predicted and ground‐truth plans and tabulated in Table 1 for all PTVs and OARs. These dosimetric indices were computed for individual plans then averaged over all plans in the test set (*n* = 68 patients). The values were reported as the mean ± 1 standard deviation. For the targets, the average difference of predicting the *D*_99_ value was 3.15 ± 2.22 Gy (PTV_70), 4.59 ± 3.24 Gy (PTV_63), and 0.23 ± 0.16 Gy (PTV_56). For the OARs, the average error of predicting $D_{max}$ value was 2.25 ± 1.59 Gy (brainstem), 2.16 ± 1.55 Gy (spinal cord), and 0.16 ± 0.12 Gy (mandible), whereas the values for $D_{mean}$ were 0.73 ± 0.52 Gy (left parotid), 1.83 ± 1.29 Gy (right parotid), 5.05 ± 3.59 Gy (esophagus), and 2.17 ± 1.53 Gy (larynx). The overall average $D_{max}$ difference (over the brainstem, spinal cord, and mandible) was 1.43 ± 1.01 Gy, whereas the value for $D_{mean}$ (over the left parotid, right parotid, esophagus, and larynx) was 2.44 ± 1.73 Gy. The overall average difference in predicting the dosimetric indices was 2.50 ± 1.77 Gy over all PTV regions, 2.37 ± 1.68 Gy over all OAR regions, and 0.75 ± 0.53 Gy over all PTV and OAR regions. Overall, the predicted plans seem to have good agreement with ground‐truth plans. The average value of the dose *HI* for the predicted plans was 7.99 ± 1.45 versus 5.74 ± 2.95 for the ground‐truth plans, whereas the average dose *CI* value was 0.63 ± 0.17 for the predicted plans versus 0.89 ± 0.19 for the ground‐truth plans.

**TABLE 1:** Dosimetric indices (dose–volume, dose homogeneity, and dose conformity indices) used for head‐and‐neck plan evaluation on the testing data set (*n* = 68 patients)

Dosimetric index	Predicted plans	Ground‐truth plans	Dose difference
PTV_70 *D*_99_ (Gy)	63.85 ± 1.72	66.99 ± 3.27	−3.15 ± 2.22 (4.70%)
PTV_63 *D*_99_ (Gy)	56.44 ± 6.17	61.03 ± 4.62	−4.59 ± 3.24 (7.52%)
PTV_56 *D*_99_ (Gy)	53.53 ± 1.83	53.31 ± 3.15	0.23 ± 0.16 (0.43%)
Brainstem $D_{max}$ (Gy)	35.60 ± 7.26	33.35 ± 9.23	2.25 ± 1.59 (6.32%)
Left parotid $D_{mean}$ (Gy)	34.74 ± 12.04	34.01 ± 13.15	0.73 ± 0.52 (2.16%)
Right parotid $D_{mean}$ (Gy)	36.10 ± 12.96	34.27 ± 13.40	1.83 ± 1.29 (5.33%)
Spinal cord $D_{max}$ (Gy)	37.75 ± 5.40	35.56 ± 5.28	2.16 ± 1.55 (6.01%)
Esophagus $D_{mean}$ (Gy)	42.85 ± 8.23	37.81 ± 6.91	5.05 ± 3.57 (13.36%)
Larynx $D_{mean}$ (Gy)	49.49 ± 12.87	47.32 ± 13.23	2.17 ± 1.53 (4.59%)
Mandible $D_{max}$ (Gy)	70.20 ± 4.45	70.37 ± 5.60	−0.16 ± 0.12 (0.23%)
All $D_{mean}$ (Gy)	40.79 ± 6.80	38.35 ± 6.22	2.44 ± 1.73 (6.36%)
All $D_{max}$ (Gy)	47.85 ± 19.39	46.42 ± 20.77	1.43 ± 1.01 (2.80%)
All *D*_99_ (Gy) or all PTVs (Gy)	57.94 ± 5.32	60.44 ± 6.86	−2.50 ± 1.77 (4.14%)
All OARs (Gy)	43.82 ± 12.75	41.81 ± 13.48	2.01 ± 1.42 (4.80%)
All PTVs and OARs (Gy)	48.06 ± 12.70	47.40 ± 14.58	0.65 ± 0.46 (1.38%)
*HI*	7.99 ± 1.45	5.74 ± 2.93	2.26 ± 1.60 (39.40%)
*CI*	0.63 ± 0.17	0.89 ± 0.19	−0.27 ± 0.19 (30.34%)

Results were reported in the form of mean ± 1 standard deviation. *D*_99_ is the dose to 99% of the volume or the minimum dose received by 99% of the target, $D_{mean}$ is the mean dose to a structure, $D_{max}$ is the maximum dose to a structure, *HI* is the homogeneity index for PTV_70, *CI* is the conformity index for PTV_70. A negative (−) percentage difference implies the predicted plan dose was lower.

Abbreviation: OAR, organs at risk; PTV, planning target volume.

**FIGURE 6:**
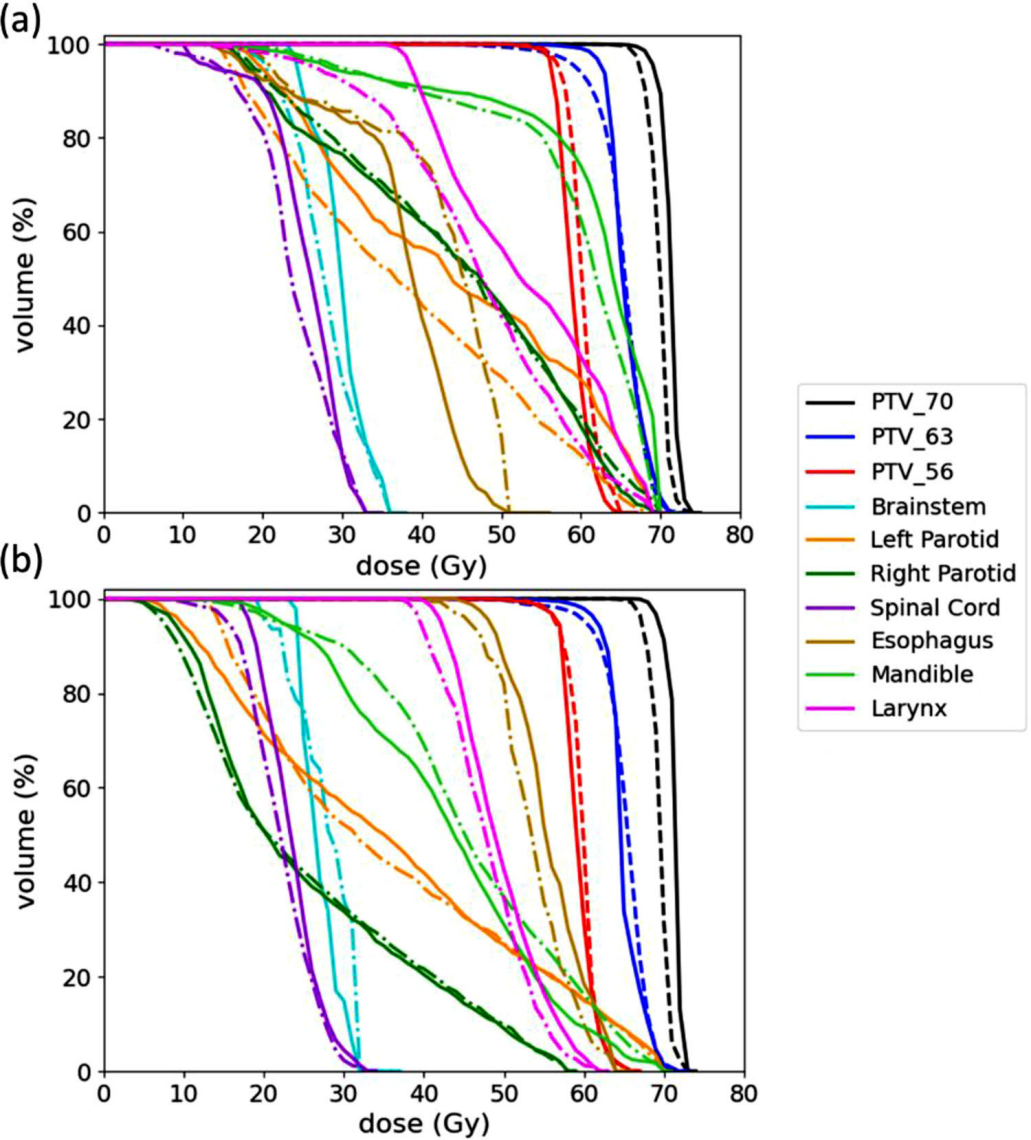
The DVHs of the predicted plans (dashed line) overlaid on the DVHs of the ground‐truth plans (solid line) for two sample patients (a) and (b) in the test set. The DVHs of the target volume structures (PTV_70, PTV_63, and PTV_56) and OAR structures (brainstem, left parotid, right parotid, spinal cord, mandible, esophagus, and larynx) are plotted in different colors as shown in the legend. DVH, dose–volume histogram

The DVH curve comparisons between the predicted and ground‐truth plans are shown in Figure 6 for two sample patients of the test set. The curves show that the targets (PTV_70, PTV_63, and PTV_56) and OAR (brainstem, left parotid, right parotid, spinal cord, mandible, esophagus, and larynx) structures of both plans are overlapping. Overall, the predicted DVHs of PTVs and OARs are similar to the ground‐truth ones.

### 3.3 Features visualization

**FIGURE 7:**
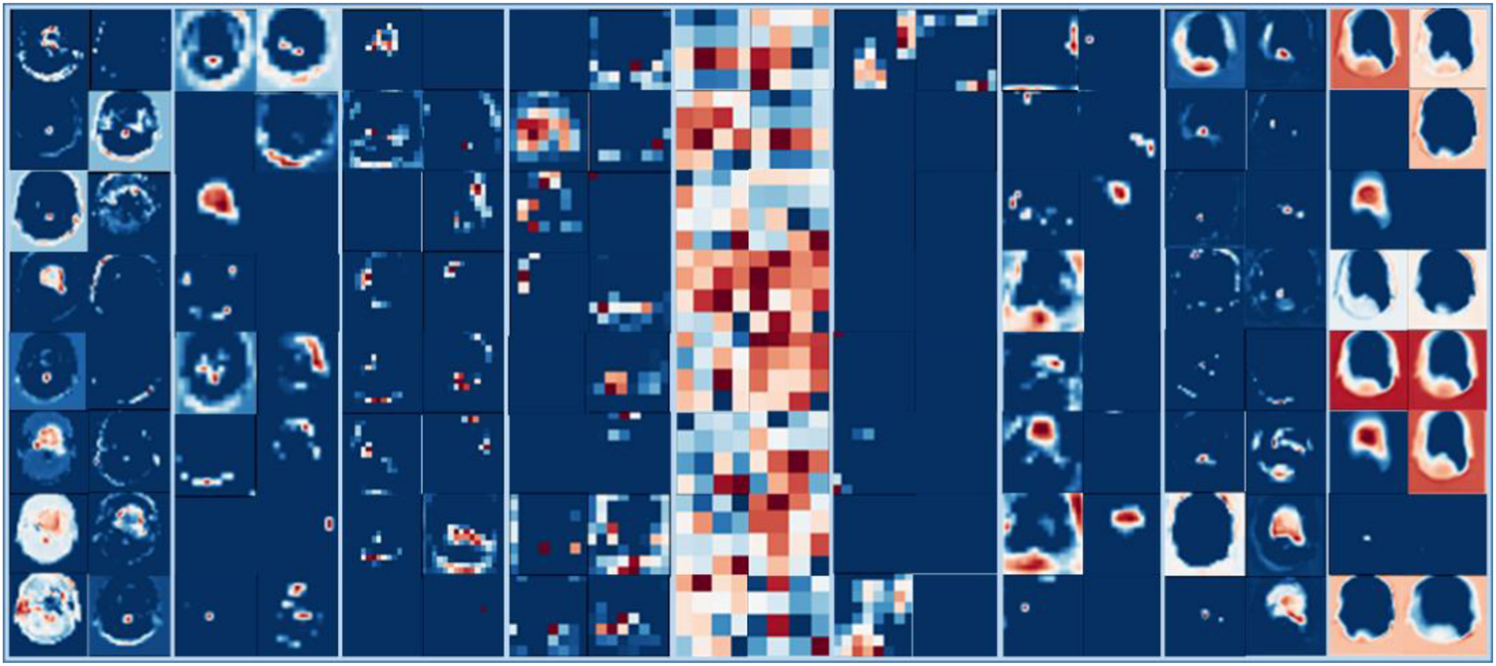
Visualizing example learned features at multiscale resolution levels of the attention‐gated U‐Net model. Each column represents an example set of 16 extracted features. The feature set in the most left column is extracted after the convolutional operations of the first hierarchy level in the encoder, representing low‐level feature maps that encode general patterns in the images. The column at the middle shows the extracted features after the convolutional operations of the last hierarchy level in the encoder (latent representation space), representing high‐level feature maps that encode task‐specific patterns in the images. The last column illustrates learned and reconstructed targeted images in the decoder

Feature map visualization helps to understand the decision basis of the convolutional models and convey that information to the human. As shown from Figure 7, during the model training process it learns different sets of features at different multiscale resolution levels. The earlier convolutional layers in the network encode low‐level features (voxel‐rich information) (e.g., edges, contrast, and enhancement texture), and as it is going deeper the model progressively transforms to learn higher level features (attribute‐rich information).

## 4 DISCUSSION

KBP methods using deep learning have been introduced for radiotherapy dose distribution prediction to reduce the planning time and maintain consistent high‐quality plans. This study proposes a novel KBP method that uses an attention‐gating mechanism on a 3D U‐Net architecture for head‐and‐neck volumetric dose prediction. The attention gates could improve efficiency and facilitate model convergence by reducing the redundancy within the network. The proposed method in this study utilizes patient volumetric anatomical information (3D CT data and contour structures) as inputs to predict the corresponding 3D dose distribution.

The data set used in this study is publicly available, which would allow other researchers to validate the proposed model performance and the reported results. This study was designed to mimic the planning environment of the dosimetrist; therefore, the input data for the proposed model include the CT image data and contours. Willems et al.^47^ reported that the performance of the dose prediction model improves when both CT and contours are used as input. We preprocessed the data by applying data normalization to speed up the training and the convergence. Consequently, we did not include batch normalization layers in the proposed network architecture. To reduce the likelihood of model overfitting and improve the model generalizability, we implemented a dropout strategy with varying rates and an early‐stopping regularization technique. As a result, the learning curves of the training and validation (Figure 2) exhibited good‐fitting behaviors. The curves have also shown the model's generalizability to new data.

The experimental results show that attention‐gated U‐Net can accurately predict head‐and‐neck dose distributions with a similar appearance to the ground‐truth dose distributions (Figures 3 and 4). The quantitative analysis of the results showed that it could predict the dose in the body contour with an average MAE value of 2.43 Gy (3.5% relative to the prescription dose), which is superior to the baseline U‐Net (MAE = 2.81 Gy) (Figure 5). In addition to the global assessment, the proposed model was also achieved lower MAE in the PTV and OAR local regions such as the left parotid (4.24 Gy [attention U‐Net] vs. 4.53 Gy [baseline U‐Net]), the mandible (4.76 Gy [attention U‐Net] vs. 4.99 Gy [baseline U‐Net]), larynx (4.50 Gy [attention U‐Net] vs. 4.92 Gy [baseline U‐Net]), PTV_70 (2.49 Gy [attention U‐Net] vs. 2.65 Gy [baseline U‐Net]), and PTV_63 (2.90 Gy [attention U‐Net] vs. 3.51 Gy [baseline U‐Net]). However, the model tends to report marginally higher MAE in the brainstem (2.97 Gy [attention U‐Net] vs. 2.92 Gy [baseline U‐Net]), right parotid (4.62 Gy [attention U‐Net] vs. 4.22 Gy [baseline U‐Net]), spinal cord (3.35 Gy [attention U‐Net] vs. 2.96 Gy [baseline U‐Net]), esophagus (6.58 Gy [attention U‐Net] vs. 5.11 Gy [baseline U‐Net]), and PTV_56 (2.33 Gy [attention U‐Net] vs. 2.25 Gy [baseline U‐Net]). Overall, these results demonstrate the superiority of the attention‐gated U‐Net over the standard U‐Net.

Besides comparing the proposed model performance to a baseline U‐Net model (similarly trained and evaluated in this study), we also made a rough comparison with the results of the top‐ranked state‐of‐the‐art methods in the OpenKBP—2020 AAPM Grand Challenge competition. In this case, it should be noted that the assessment process is slightly different even though they all were trained on the same data pool. One of these differences is that the proposed model in this study was not evaluated on the exact data set due to the random nature of splitting the data into training/validation/testing cohorts. Furthermore, we assessed the dose prediction error (e.g., MAE) over voxels within the body contour, whereas the OpenKBP methods were evaluated over a specific voxels’ mask for consistent comparison of participating methods in the competition. Consequently, the computed average MAE values may differ. The rough comparison results showed that the proposed model performance (MAE = 2.43 Gy) was competitive to that proposed by Liu et al.^38^ (MAE = 2.31 Gy) using Cascade 3D U‐Net, which achieved the first place in the OpenKBP competition. Moreover, it was superior to the second‐ranked method proposed by Gronberg et al.^37^ (MAE = 2.56 Gy) using 3D dense dilated U‐Net, and the third‐ranked method proposed by Zimmermann et al.^41^ (MAE = 2.62 Gy) using GAN. Also, a rough comparison was made to other dose prediction methods in the literature where the assessment process is typically similar to what we used in this study with the MAE calculated over all voxels within the body contour. The results demonstrated the outperformance of our model (MAE = 3.5%, relative to the prescription dose) over the Chen et al.^33^ method using Res‐Net‐101 (MAE = 5.3% relative to the prescription dose).

The DVHs analysis showed good agreement between the predicted and ground‐truth plans (Table 1). The average difference in *D*_99_ index within all regions of interest (PTVs and OARs) achieved by our proposed method (0.75 Gy) was much lower than that reported by the Liu et al.^38^ method (2.5 Gy). The dose homogeneity results in the target (PTV_70) for the predicted plans (*HI* = 7.99) were slightly higher than those calculated for the ground‐truth plans (*HI* = 5.74). This means that the model tends to overestimate the *HI* value and predict a less homogeneous dose for the target. Dose conformity results showed that the predicted plans (*CI* = 0.63) have a lower conformal dose in the PTV than the ground‐truth plans (*CI* = 0.89). The proposed model overestimates the dose in the target (PTV_70), and re‐normalizing the dose to 95% of the prescription dose instead of 100% may improve the *CI*. In this study, we did not use the number of plans that satisfied the clinical criteria for head‐and‐neck planning as it is not always necessary that a clinically acceptable plan should meet all criteria. The DVH curves (Figure 6) of most structures are almost overlapping. One possible way to improve the reported results in this study is by reducing the voxel size of the input data to minimize structures overlapping due to coarse resolution; however, this will require more memory and computational power.

Implementation of the KBP in radiotherapy treatment planning would resolve some inherent shortcomings existing with the current treatment planning practice, for example, deliverable dose to OARs is unknown a priori. In addition, the heterogeneity of clinical practices can lead to variation in the achievability of the planning goals and the plan quality. The core goal is to discover the best capabilities of the deep learning techniques to develop dose prediction models able to closely replicate the manually produced clinical plans (not necessarily predicting better plans). Of course, if the dose prediction models manage to improve upon the clinical plans, it would be even better. The proposed model in this study has more clinical universality due to its ability to handle challenging head‐and‐neck cases with multiple targets prescribed to different doses. Furthermore, the proposed model utilizes 3D deep convolutional network architecture where it considers the contextual information of adjacent slices in predicting more accurate and representative 3D dose distributions compared to using only 2D images. This is due to fact that dose distributions are not only related to the current slice but also associated with adjacent slices. The 3D model predicts more realistic dose distributions than the 2D network with smoother dose gradients across the longitudinal axis.

The limitations of this study could be highlighted as follows. *First*, we only used IMRT data for training our network, and it was only evaluated on head‐and‐neck anatomy. As a result, we cannot confirm whether an IMRT dose prediction model can be applied for VMAT or works successfully on other anatomical treatment regions. We recommend retraining the model if it is intended to be used on other treatment sites’ data to obtain satisfactory performance. *Second*, the prediction model in this study was trained on a fixed beam configuration; however, in routine clinical practices, beam orientations could broadly vary from patient to patient, from planner to planner, and from institution to institution. *Third*, the proposed model in this study provides predicted clinically acceptable dose distributions only, which require a further optimization step to be converted into a deliverable plan. Future investigations will focus on converting the predicted 3D dose distributions into an executable clinical plan by determining the delivery parameters.

## 5 CONCLUSION

We developed a novel KBP method by combing the strength of a 3D U‐Net and the attention‐gating mechanism to accurately predict 3D dose distributions for head‐and‐neck cancer patients treated with IMRT. The attention‐gating mechanism focuses only on the relevant anatomy that improves the learning; thus it would improve the prediction performance. In addition, the 3D structure of the proposed model in this study allows considering the contextual information between the adjacent image slices for more accurate predictions. The results demonstrated that the attention‐gated 3D U‐Net model is capable of predicting accurate dose distributions that closely replicate the ground‐truth plans. The proposed model exhibited superior performance to a baseline 3D U‐Net that was similarly trained and evaluated on the same data set. Compared to the state‐of‐the‐art methods reported in the literature, the proposed model demonstrated competitive performance to the top‐ranked method in the OpenKBP competition and was superior to the second‐ranked method. The proposed model revealed its promise for clinical applications with great potential to increase the efficiency of the clinical radiotherapy treatment planning workflow and maintain consistent plan quality. It takes a few seconds to generate a full 3D dose distribution for a new patient, making it practical for real‐time applications (e.g., online adaptive radiotherapy). The model could be used as a decision support tool for the treating physician before starting the treatment planning or as planning guidance for the planners. It can also be utilized as a part of a fully automated treatment planning pipeline by integrating the model into the plan optimization process. Furthermore, the model may be applied for quality assurance in clinical trials, where plan quality is assessed according to whether it has met the constraints.

## CONFLICT OF INTEREST

The authors have no conflict of interest to disclose.

## AUTHOR CONTRIBUTION

Alexander F. I. Osman contributed to the conception and design of the study, developing the models, drafting the revising the manuscript for important intellectual contents. Nissren M. Tamam contributed to writing and revising the manuscript. All authors contributed to manuscript revision and approved the submitted version.

## Data Availability

The data sets can be found in the OpenKBP—2020 AAPM Grand Challenge repository at https://competitions.codalab.org/competitions/23428. The code for the model developed in this study is also available as an open‐source package on GitHub respiratory at https://github.com/afiosman/attention‐aware‐3D‐UNet‐for‐RT‐dose‐prediction.
